# Green Tea, Intermittent Sprinting Exercise, and Fat Oxidation

**DOI:** 10.3390/nu7075245

**Published:** 2015-07-13

**Authors:** Daniel Gahreman, Rose Wang, Yati Boutcher, Stephen Boutcher

**Affiliations:** 1 Department of Exercise and Sport Science, Charles Darwin University, Ellengowan Drive, Casuarina, Northern Territory 0811, Australia; E-Mail: daniel.gahreman@cdu.edu.au; 2 School of Medical Sciences, Faculty of Medicine, University of New South Wales, High Street, Randwick, Sydney, New South Wales 2052, Australia; E-Mails: rose.wang589@gmail.com (R.W.); y.boutcher@unsw.edu.au (Y.B.)

**Keywords:** green tea, high-intensity exercise, epinephrine, norepinephrine, post exercise lipolysis

## Abstract

Fat oxidation has been shown to increase after short term green tea extract (GTE) ingestion and after one bout of intermittent sprinting exercise (ISE). Whether combining the two will result in greater fat oxidation after ISE is undetermined. The aim of the current study was to investigate the combined effect of short term GTE and a single session of ISE upon post-exercise fat oxidation. Fourteen women consumed three GTE or placebo capsules the day before and one capsule 90 min before a 20-min ISE cycling protocol followed by 1 h of resting recovery. Fat oxidation was calculated using indirect calorimetry. There was a significant increase in fat oxidation post-exercise compared to at rest in the placebo condition (*p <* 0.01). After GTE ingestion, however, at rest and post-exercise, fat oxidation was significantly greater (*p <* 0.05) than that after placebo. Plasma glycerol levels at rest and 15 min during post-exercise were significantly higher (*p <* 0.05) after GTE consumption compared to placebo. Compared to placebo, plasma catecholamines increased significantly after GTE consumption and 20 min after ISE (*p* < 0.05). Acute GTE ingestion significantly increased fat oxidation under resting and post-exercise conditions when compared to placebo.

## 1. Introduction

The increasing prevalence of overweight and obesity is associated with numerous cardiovascular and metabolic diseases [1,2,3]. Weight reduction treatments designed to induce fat loss include steady state exercise, appetite suppressants, and lipase inhibitors [4,5]. Energy restricted diets have also been used in an attempt to lower the body fat of obese individuals [6]. The minimal long-term effectiveness of these methods has focused attention on other fat loss strategies such as green tea ingestion [7] and participation in intermittent sprinting exercise (ISE) [8].

Green tea extract (GTE) is produced from the leaves of *camellia sinensis* [1] and contains catechins which are the predominant form of polyphenols. The major catechins are epigallocatechin gallate (EGCG), epigallocatechin (EGC), epicatechin gallate (ECG), and epicatechin (EC) [9]. EGCG is the most pharmacologically active of the catechins which typically accounts for approximately 50% of the catechin content of green tea [9]. Green tea typically contains a small amount of caffeine estimated to be about three to five percent of its dry weight [10]. Short term ingestion of tea catechins, typically one to two days before testing, has been shown to increase fat oxidation, particularly during the postprandial period, as indicated by a reduced respiratory exchange ratio (RER) during indirect calorimetry [4,11,12,13,14]. It has been suggested that the catechins in green tea increase fat oxidation through inhibition of catechol-*O-*methyltransferase, an enzyme that degrades norepinephrine, thereby prolonging the action of sympathetically released norepinephrine [15,16,17].

Steady state aerobic exercise results in small increases in plasma catecholamines [18,19]. In contrast, significantly elevated epinephrine and norepinephrine levels during 20 min of ISE in trained and untrained young women have been found [20]. The catecholamine response is an important feature of ISE as catecholamines have been shown to drive lipolysis and are largely responsible for fat release from both subcutaneous and intramuscular fat stores [21]. The significant catecholamine response to ISE may underlie the ability of regular ISE to induce greater fat loss than that occurring after regular steady state exercise. For example, Trapp *et al.* [22] compared steady state cycle exercise and ISE using a bout of ISE lasting 8 s with a 12 s recovery (20 min total) for three times per week for 15 weeks. Fat mass significantly decreased in the ISE condition compared to no reduction in subcutaneous fat after steady state exercise. A similar protocol with overweight males also resulted in significant decreases in total and visceral fat [23]. Given the important role of the neurotransmitter norepinephrine in the control of fat oxidation, it is conceivable that GTE, by inhibiting norepinephrine breakdown, may enhance fat oxidation after ISE. Another possible contributor to ISE-induced exercise recovery fat oxidation, however, could be the post exercise oxygen uptake (EPOC) in excess of that required at rest [24].

Although the combined effect of short term GTE and acute ISE on fat oxidation during post-exercise has not been examined one study has investigated the ability of GTE to elevate fat oxidation during 30 min of steady state aerobic exercise. Venables *et al.* [25] found that short term GTE ingestion increased fat oxidation by 17% compared to placebo during a 30-min continuous bout of moderate intensity cycling exercise. In contrast, it was found that one day of GTE ingestion had no effect on markers of lipolysis or fat oxidation during moderate intensity, cycling exercise, however, seven days of GTE supplementation increased markers of lipolysis but had no effect on fat oxidation [26]. As studies were similar in their design and participant characteristics, authors suggested that the addition of caffeine in the more recent study could have suppressed fat oxidation as there is a negative correlation between lactate level and fat oxidation [27].

As these results were obtained during moderate intensity aerobic exercise, it appears that the effect of short term ingestion of GTE on fat oxidation, before and after ISE, has not been investigated. Therefore, the aim of this study was to examine the effect of short term GTE ingestion and one bout of ISE on fat oxidation of untrained women. It was hypothesized that the combination of GTE and ISE, compared to ISE alone, would result in significantly greater fat oxidation during the post-exercise period.

## 2. Experimental Section

### 2.1. Participants

Fourteen untrained females (age: 21.5 ± 0.5 years; body mass: 65.7 ± 1.8 kg; BMI: 24.3 ± 0.4 kg/m^2^; maximal oxygen consumption ($\dot{V}$O_2max_): 32.1 ± 1.7 mL/kg/min) were recruited to participate in the study. All women were healthy judging from response to a general health survey and all gave written consent for participation. Exclusion criteria included women who were pregnant and regular caffeine (≥2 cups coffee/day) or green tea drinkers (≥2 cups tea/day). The study was approved by a university human research ethics committee.

### 2.2. Preliminary Testing

After an overnight fast of approximately 10 h participants attended the laboratory between 7:00 and 9:00 am where baseline anthropometric measurements, fasting blood sugar level and lipid profile assessment, a $\dot{\text{V}}$O_2max_ test, maximum power output assessment, and induction to ISE training was carried out. Anthropometric measures were height, body mass, and BMI. A 23-gauge butterfly needle was inserted into an antecubital vein and blood samples were placed in 10 mL EDTA and 4 mL heparin sodium vacutainers (Becton Dickinson, Plymouth, UK). Blood lipid levels were assessed immediately (Cholestech LDX, Hayward, CA, USA). The laboratory was maintained at a constant ambient air temperature of 22 °C to 23 °C.

An electrically braked, computer-controlled Monark 839E ergometer (Monark, Vansbro, Sweden) linked to a ParvoMedics TrueMax 2400 metabolic cart (ParvoMedics, Sandy, UT, USA) was used to assess $\dot{\text{V}}$O_2max_. Participants were required to maintain a cycling cadence of approximately 70 revolutions per minute (RPM) that was displayed on a computer screen and monitored by an experimenter. Each participant completed a 3-min warm-up at 30 watts (W) after which load was increased by 15 W per min until exhaustion. Heart rate (HR) was recorded continuously using a Polar Watch S810i (Polar Electro, Kempele, Finland). $\dot{\text{V}}$O_2max_ was considered to be maximal when at least two of the following conditions were achieved: (1) leveling of $\dot{\text{V}}$O_2_ even with an increase in workload; (2) a RER ≥ 1.10; and (3) reduced pedaling speed despite encouragement. Range of time duration for the $\dot{\text{V}}$O_2max_ test was 8 min 58 s to 12 min 15 s. Participants then practiced the ISE protocol for 10 min [28] on a Monark 839E ergometer which consisted of repeated bouts of 8-s sprint cycling at 60% of maximum power output and 12 s recovery at 20% of maximum power output. Exercise was accompanied by a tape which prompted the start and finish of sprint and recovery and the pedal cadence (110 RPM) during the sprint and recovery (40 RPM) phases.

### 2.3. General Study Design

A double-blinded crossover design involving women completing two exercise sessions with either GTE or placebo was used. A wash-out period of four weeks separated sessions. To standardize testing both exercise sessions were performed during the luteal phase of consecutive menstrual cycles at the same time of day. Determination of luteal phase was achieved through questioning each participant before arranging each testing session. All women reported that they did not use oral contraceptives.

### 2.4. Diet and Capsule Content

Participants recorded a food diary for 3 days prior to the first session and were requested to follow the same diet before the second session. The day before each exercise session, participants ingested one capsule containing either GTE or cellulose with breakfast, lunch, and dinner. Order of GTE and placebo was counter-balanced. After approximately 10 h of overnight fasting, the fourth capsule was consumed on the morning of the next day, 50 min before baseline, and 90 min before exercise [25]. The three GTE capsules consumed the day before exercise contained a total of 562.5 mg polyphenols and 375 mg, whereas the one GTE capsule consumed on the exercise day, 90 min before exercise, contained 187.5 mg polyphenols and 125 mg EGCG. Green tea catechins (EGCG, EGC, and EC) have been shown to peak in the blood between 1.3 h and 1.6 h [29]. The GTE capsule (GNC, Pittsburgh, PA, USA) contained 250 mg of *camellia sinesis* extract (187.5 mg polyphenols, 125 mg EGCG) [30]. Each GTE capsule contained 20 mg of caffeine. The placebo capsule contained 500 mg cellulose. All participants were reminded by text message to ingest GTE or placebo capsules the day before and on the day of exercise and all reported that they had ingested the capsules.

### 2.5. Experimental Protocol

The second and third exercise sessions consisted of three phases: (1) rest, (2) ISE, and (3) post-exercise. Participants reported to the laboratory between 7:00 and 9:00 am after the approximate 10-h fast, having ingested the fourth GTE capsule 50 min previously. A 22-gauge cannula (Becton Dickinson, Plymouth, UK) was inserted into each participant’s forearm antecubital vein and a 3-way stopcock (Becton Dickinson, Plymouth, UK) was used for repeated blood sampling. The 3-way stopcock line was kept patent by flushing with 0.9% isotonic saline (Pfizer, New York, NY, USA). A resting blood sample was taken at least 30 min after insertion of the cannula [31]. Blood samples, which were centrifuged for 10 min at 3000 RPM after rest, exercise, and post-exercise, were collected in a vacutaneur containing EDTA. Blood plasma was extracted and stored at −86 °C for catecholamine and glycerol analysis.

### 2.6. Rest

During the first 10 min of rest each participant laid on a plinth and rested whilst no data were collected. Data were then collected for the next 40 min. The first 10 min of the 40-min data collection period were eliminated and the remaining 30 min of data were screened so that data affected by behavior such as coughing and posture change could be excluded. $\dot{\text{V}}$O_2_, $\dot{\text{V}}$CO_2_, energy expenditure (EE), and RER were measured for 30 min using a TrueOne 2400 Canopy system (ParvoMedics, Sandy, UT, USA). EE was calculated by using the Weir equation [32]. HR was recorded continuously using the Polar watch*.* At the end of the rest period blood lactate levels were assessed (Accutrend Lactate monitor, Roche, Germany). Support for the validity of resting metabolic rate calculated by the Parvomedics system has been previously provided [33].

### 2.7. Interval Sprinting Exercise and Post Exercise

After a 5-min warm-up at 30 W, participants immediately completed 20 min of ISE on a Monark Ergomedic 839E ergometer linked to the metabolic cart. Pedal cadence was paced at 110 RPM during the sprint phase and 40 RPM during the recovery phase. Pedal resistance for the sprint phase was calculated as 60% of each participant’s maximal power output (W_max_) determined from prior testing and 20% for the recovery phase. Pedal cadence was monitored throughout exercise and recorded for both phases at minutes 5, 10, 15, and 20. In the 20-min ISE session, sixty 8-s/12-s bouts totaling 8 min of sprinting and 12 min of easy pedaling recovery were performed followed by a 5-min cool-down at 30 W. RPM for sprinting recorded every 5 min, whereas HR was recorded every beat and then averaged each 15 s. Gas sampling was averaged every 15 s throughout the whole 20 min of exercise. Blood was sampled at 7 min, 14 min, and 20 min during the exercise recovery pedaling period and blood samples from min 7 and 20 were assayed immediately for lactate. Lactate was also analysed during post-exercise at min 30 and 75. Glycerol levels were measured using blood collected before exercise; 7 min, 14 min, and 20 min into exercise; and 15 min, 30 min, 45 min, 60 min, and 75 min post-exercise. Epinephrine and norepinephrine levels were assessed using blood collected before exercise; after completing 20 min of ISE; and 20 min post-exercise. RER was averaged at 5-min stages. RER was analysed during the 30 min to 75 min period post-exercise because with changing blood lactate concentrations (e.g., immediately after exercise) the bicarbonate concentration is also changing which results in CO_2_ production that influences RER without necessarily representing the true quotient [34]. During the 30 min to 75 min period post-exercise lactate levels were stable. Rating of perceived exertion (RPE) was recorded every 5 min during exercise [35].

**Figure 1:**
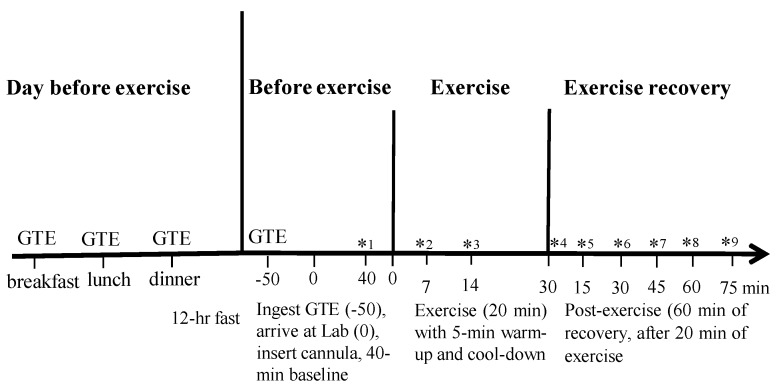
Diagrammatic representation of the study design. * indicates blood collection. Lactate assessed at *^1^, *^2^, *^4^, *^6^, *^9^; catecholamines at *^1^, *^4^, *^5^, and glycerol at *^1–^*^9^.

After exercise, participants rested for 15 min allowing time for gas and volume calibration before undertaking the 1-h post-exercise period under the ventilated canopy. The 1-h post-exercise period started at minute 15 after exercise and finished at minute 75 (Figure 1). HR was monitored continuously and blood was sampled every 15 min during minutes 15 to 75 during post-exercise and was assessed for lactate at 30 min and 75 min post-exercise (Figure 1). Figure 1 illustrates the time line of the study.

### 2.8. Blood Variables and Oxidation Rates

Glycerol was measured using the Free Glycerol Determination (FG0100) reagent assay kit (Sigma Aldrich) and Glycerol Standard (G7793). The degree of enzymatic turnover of the substrate was determined by dual wavelength absorbance measurement at 450 nM and 540 nM. The coefficient of variation (CV) for glycerol was 7.8%. Norepinephrine and epinephrine were measured using mass spectrometry with a 5973N Mass Selective Detector, coupled to a 6890N gas chromatograph, and an SGE Forte BPX5 × 0.25 ID_×_ 0.25 micron column [36]. Accuracy and precision were determined by analysis of spiked serum samples at low, medium, and high nM concentrations of norepinephrine and epinephrine in triplicate on 7 separate days. Serum norepinephrine recoveries at 20, 100, and 500 nM were above 95% and inter-day average CV was 4.28%; similarly, epinephrine recoveries at 2 nM, 10 nM, 50 nM were above 99% and inter-day average CV 5.88% (*n* = 21 for each concentration level). Average intra-day CV was 2.02% for norepinephrine and 2.26% for epinephrine. Average intra-day recoveries of 98.6% and 102% were obtained for norepinephrine and epinephrine respectively.

Fat and carbohydrate oxidation rates (g/min) were calculated using the following Equations [37]:

$$\mathrm{Fat}\mathrm{oxidation}\;(\mathrm{1.695}\times \dot{\text{V}}O_{2})-(\mathrm{1.701}\times \dot{\text{V}}{\mathrm{CO}}_{2})$$

$$\mathrm{Carbohydrate}\mathrm{oxidation}\;(\mathrm{4.585}\times \dot{\text{V}}O_{2})-(\mathrm{3.226}\times \dot{\text{V}}{\mathrm{CO}}_{2})$$

When assessing fat oxidation rate only the values at rest and during minutes 30 to 75 during post-exercise were used because RER does not represent substrate utilization when blood and tissue lactate concentrations are changing [34]. Lactate levels were assessed using whole blood obtained from the indwelling cannula.

### 2.9. Statistical Analysis

Data were analyzed using SPSS 20.0 (SPSS Inc., Chicago, IL, USA). A two-factor (time × condition) repeated measures ANOVA was used to compare differences across time and condition. The Mauchly sphericity test was used to test for homogeneity of covariance for within subject factors. The Greenhouse-Geisser test was used to correct for non-homogenous values. When repeated measures ANOVA interactions were significant, adjusted Bonferroni *post hoc* tests were also performed. Data are presented as mean ± SEM and significance was set at *p* < 0.05.

## 3. Results

### 3.1. Blood Testing

Total cholesterol (4.26 ± 0.2 mmol/L), high density lipoprotein (1.45 ± 0.1 mmol/L), low density lipoprotein (2.76 ± 0.3 mmol/L), triglyceride (0.72 ± 0.1 mmol/L), and blood glucose levels (4.8 ± 0.4 mmol/L) were in the normal range for women of this age.

### 3.2. Workload and Exercise Intensity

There were no significant differences in mean power output, RPE, lactate levels, RPM (Table 1), and HR levels between the GTE and placebo trials (Table 2).

**Table 1:** Mean power output, rating of perceived exertion, and lactate response to the sprinting and recovery components of the intermittent sprinting exercise for the placebo and green tea conditions (mean and SEM).

	Placebo	GTE
Mean power output (W) during 8 s sprint	99.2 ± 5.7	99.4 ± 6.1
Revolutions per minute during 8 s sprint	111.2 ± 1.1	111.3 ± 1.5
Pedal resistance (kg) during 8 s sprint	0.9 ± 0.10	0.9 ± 0.10
Mean power output (W) during 12 s recovery	33.4 ± 3.4	33.8 ± 3.7
Revolutions per minute during 12 s recovery	37.2 ± 0.66	38.4 ± 0.70
Pedal resistance (kg) during 12 s recovery	0.9 ± 0.10	0.9 ± 0.10
Rating of perceived exertion throughout	13.8 ± 0.7	13.4 ± 0.5
Lactate (mmol/L) at 7 min	3.8 ± 0.3	4.1 ± 0.2
Lactate (mmol/L) at 20 min	5.3 ± 0.5	5.1 ± 0.3

GTE: green tea extract.

**Table 2:** Response at rest and during and after intermittent sprinting exercise in the green tea and placebo conditions (mean and SEM).

Variable	Condition	Rest	Exercise	Post Exercise		
				35 min	55 min	75 min
Heart rate (bpm)	GTE	65.50 ± 3.25	156.31 ± 2.7	77.21 ± 3.7	73.71 ± 3.6	74.00 ± 3.6
	Placebo	64.84 ± 2.8	153.95 ± 2.7	75.95 ± 3.8	72.68 ± 3.5	71.28 ± 3.6
$\dot{\text{V}}$O_2_ (L/min)	GTE	0.209 ± 0.005	1.513 ± 0.047	0.235 ± 0.005 *	0.217 ± 0.005 *	0.217 ± 0.005 *
	Placebo	0.207 ± 0.005	1.463 ± 0.043	0.225 ± 0.005	0.209 ± 0.005	0.208 ± 0.005
$\dot{\text{V}}$CO_2_ (L/min)	GTE	0.173 ± 0.007	1.438 ± 0.041	0.176 ± 0.006	0.168 ± 0.005	0.177 ± 0.006
	Placebo	0.179 ± 0.005	1.403 ± 0.035	0.181 ± 0.005	0.172 ± 0.006	0.177 ± 0.005
RER	GTE	0.83 ± 0.03 *	0.95 ± 0.03 *	0.75 ± 0.03 *	0.78 ± 0.02 *	0.81 ± 0.02 *
	Placebo	0.86 ± 0.03	0.97 ± 0.02	0.80 ± 0.03	0.82 ± 0.03	0.85 ± 0.03
EE (kcal/min)	GTE	1.01 ± 0.08	7.48 ± 0.24	1.11 ± 0.08	1.03 ± 0.08	1.04 ± 0.08
	Placebo	1.00 ± 0.05	7.25 ± 0.21	1.08 ± 0.05	1.00 ± 0.08	1.01 ± 0.08

GTE: green tea extract; $\dot{\text{V}}$O_2_: oxygen uptake; $\dot{\text{V}}$CO_2_: carbon dioxide; EE: energy expenditure; RER: respiratory exchange ratio. * Significantly different compared to placebo, *p* < 0.01.

### 3.3. Lactate

Blood lactate levels were similar at rest during the GTE (1.76 ± 0.17 mmol/L) and placebo (1.67 ± 0.13 mmol/L) conditions. Lactate levels were significantly increased, *p <* 0.05, during exercise in both conditions (Table 1). During post-exercise (30 min and 75 min) lactate levels were similar for both the GTE (2.5 ± 0.24 mmol/L; 2.1 ± 0.15 mmol/L) and placebo (2.5 ± 0.25 mmol/L; 2.0 ± 0.20 mmol/L) conditions.

### 3.4. Respiratory Exchange Ratio (RER)

During the resting period, before exercise, RER was significantly decreased, *p <* 0.05, after GTE ingestion compared to placebo (Table 2). There was also a significant condition main effect (*p <* 0.01) with RER being significantly decreased throughout the 60 min post-exercise period in the GTE compared to the placebo condition (Table 2).

### 3.5. Fat Oxidation 

**Figure 2:**
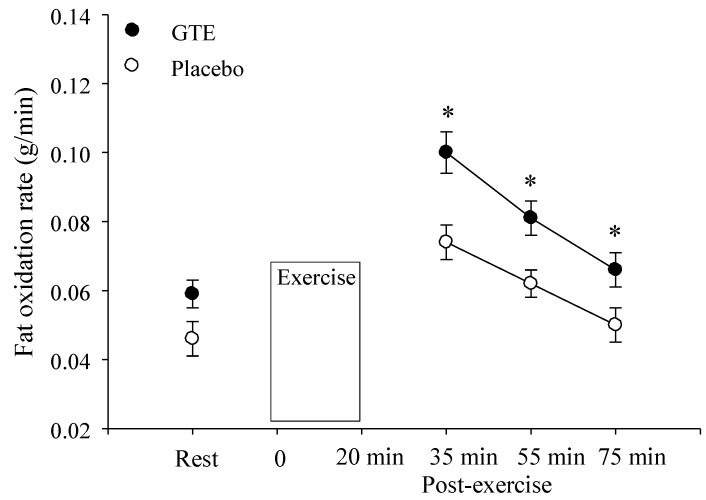
Fat oxidation at rest and after intermittent sprinting exercise with either placebo or green tea extract (GTE) ingestion. * Significantly greater compared to placebo, *p* < 0.05.

During the resting period, before exercise, fat oxidation significantly increased by 24% (*p <* 0.01) after GTE ingestion (0.059 ± 0.004 g/min) compared to placebo (0.045 ± 0.005 g/min; Figure 2). In the 30 min to 75 min period during post-exercise there was a significant condition main effect (*p* < 0.01). Fat oxidation rate was significantly increased by 29% (0.069 ± 0.006 g/min) from 35 min to 75 min during post-exercise in the GTE condition compared to the fat oxidation response during placebo (0.049 ± 0.004 g/min; Figure 2).

### 3.6. $\dot{V}$O_2_, $\dot{VC}$O_2_, and Energy Expenditure

Oxygen consumption was significantly higher (*p* < 0.01) during post-exercise in the GTE condition, whereas $\dot{\text{VC}}$O_2_ levels were similar (Table 2). Energy expenditure (EE) was similar throughout testing in the GTE and placebo conditions (Table 2).

### 3.7. Glycerol and Catecholamine Levels

**Figure 3:**
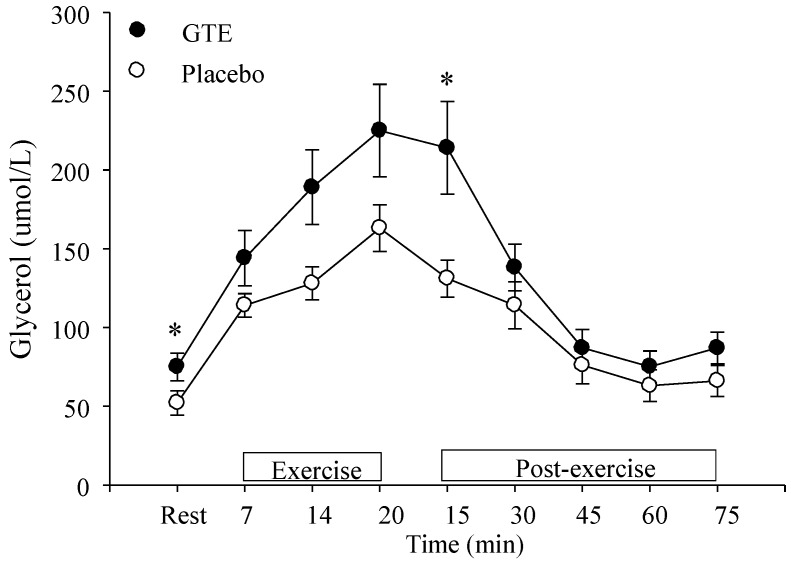
Glycerol levels at rest, during, and after intermittent sprinting exercise with either placebo or green tea extract (GTE) ingestion. * Significantly greater compared to placebo, *p* < 0.05.

At rest and at minute 15 during post-exercise GTE ingestion brought about significantly higher (*p* < 0.05) plasma glycerol concentrations compared to placebo (Figure 3).

**Figure 4:**
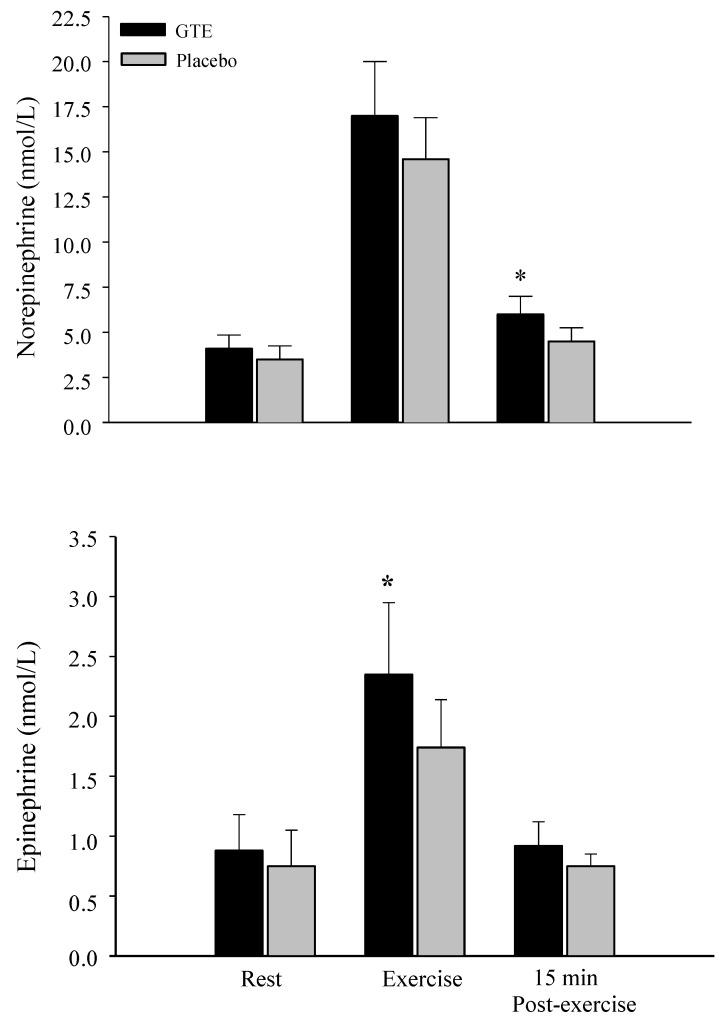
Epinephrine and norepinephrine levels at rest, during, and after intermittent sprinting exercise with either placebo or green tea extract (GTE) ingestion. * Significantly greater compared to placebo, *p* < 0.05.

Epinephrine plasma levels were significantly increased (*p* < 0.05) during exercise after GTE compared to the placebo condition (Figure 4). GTE ingestion also brought about significantly higher (*p* < 0.05) plasma norepinephrine concentrations compared to placebo at 15 min during post-exercise (Figure 4).

## 4. Discussion

The combined effect of one bout of intermittent sprinting exercise (ISE) and short term ingestion of green tea extract (GTE) on fat oxidation of untrained women was examined. During pre-exercise rest GTE ingestion significantly increased fat oxidation. Fat oxidation levels were significantly higher throughout minutes 30 to 75 during post-exercise. Also plasma glycerol levels at rest and after ISE were significantly higher after GTE consumption compared to the placebo condition. Plasma epinephrine levels showed a significant increase during ISE compared to placebo, whereas norepinephrine levels were significantly higher 15 min during post-exercise after GTE ingestion.

The ability of short term GTE ingestion to enhance fat oxidation at rest has been previously demonstrated [4,11,12,13,14]. Dulloo *et al.* [11] showed that GTE compared to placebo increased resting fat oxidation by 9.9%, whereas Rumpler *et al.* [14] found that ingestion of oolong tea increased fat oxidation by 12%. Some studies have found an elevation in resting energy expenditure (EE) after short term GTE ingestion [10,14]. Women in the present study demonstrated a 24% increase in fat oxidation during rest after short term GTE consumption (Figure 2), however, we did not find the elevation in resting EE after GTE consumption that has been demonstrated by others [10,14]. Lack of elevation in resting EE was probably because GTE consumption appears to exert more of an effect on postprandial EE. For example, Dulloo *et al.* [11] found increases in EE during a 24-h period during which three meals were consumed but no EE increase was found during sleep. The majority of studies examining GTE have recruited male participants [10,11,14,25,38] thus the present results demonstrate that GTE consumption also significantly elevates resting fat oxidation of females. With that said the blood levels of the different catechins contained in the GTE were not assessed, thus, a limitation of the study is that it cannot be shown that GTE directly influenced fat oxidation. Also another study limitation concerns the assessment of active nutrient content contained in the green tea capsules which was not independently analyzed. Also reminding participants to consume their capsules by text message and not using a more objective method to verify capsule ingestion was another limitation. Finally, a four-week wash-out period separated sessions to standardize testing during the luteal phase of consecutive menstrual cycles at the same time of day. This design controlled for menstrual cycle influences on fat oxidation but could have allowed for physical fitness changes.

The higher plasma glycerol levels at rest and during post-exercise indicate that GTE compared to placebo results in enhanced markers of lipolysis. These results support prior research that manipulated blood free fatty acid levels by administering either nicotinic acid or heparin during a hard 90-min bout of moderate and high-intensity exercise [39]. Results showed that during 6 h of exercise recovery plasma-derived free fatty acids was the major fuel source driving enhanced fat oxidation. Although plasma free fatty acid availability changed significantly, no marked change in intramuscular triglyceride (IMTG) concentration was detected. Therefore, after a bout of moderate and high-intensity exercise that resulted in blood lactate levels in excess of 5 mmol/L free fatty acids were found to drive fat oxidation. For shorter, higher intensity exercise such as intermittent sprinting, however, IMTG stores are thought to make more of an influence on whole body fat oxidation as subcutaneous adipose fat stores do not contribute significantly to high-intensity sprinting exercise [19]. The sprinting component (fast pedaling) of ISE appears to be mainly fuelled by creatinine-phosphate and anaerobic glycolysis [40]. Oxygen bound to myoglobin also appears to make a small contribution to energy production during sprinting [40], however, the major role of aerobic metabolism appears to be the resynthesis of creatinine-phosphate during the recovery sprint periods [40]. During the slow pedaling recovery component enhanced lipid utilization is also believed to occur [41,42]. The brief recovery periods of ISE are thought to prevent complete glycogen resynthesis, therefore, the glycogen depletion accompanying continuous sprinting is thought to impede glycolysis resulting in increased oxidation of IMTG [42]. Therefore, enhanced lipid oxidation could occur with a higher participation of lipolysis to the aerobic component during exercise recovery. Although an increase in fat oxidation was observed during exercise after short term GTE ingestion compared to placebo these values are not valid indicators of mitochondrial O_2_ consumption and CO_2_ production because $\dot{\text{V}}$CO_2_ assessed by indirect calorimetry is influenced by bicarbonate pool depletion [34]. Consequently, using the O_2_ and CO_2_ response during sprinting exercise to reflect nutrient usage typically overestimates carbohydrate and underestimates fat oxidation [34]. Blood lactate concentrations were not changing during minutes 30 to 75 during exercise recovery, thus fat oxidation rate was assessed during this period. That $\dot{\text{VC}}$O_2_ levels were similar and consistent during this period during exercise recovery for both GTE and placebo conditions also support the notion that the bicarbonate pool was stable [34].

Venables *et al.* [25] investigated the acute combined effects of GTE and steady state exercise (30 min cycling at 60% $\dot{\text{V}}$O_2max_) using O_2_ and CO_2_ response to establish fat oxidation levels. GTE significantly elevated fat oxidation during steady state exercise by 17% relative to placebo. Results of the Venables *et al.* [25] study show that GTE ingestion can further enhance markers of lipolysis during steady state exercise even though exercise alone also brought about an increase in lipolysis. The effect of endurance training and longer term GTE supplementation on fat oxidation during exercise has also been examined in a 10-week intervention with untrained males [43]. Results indicated that regular GTE ingestion, together with moderate intensity aerobic exercise, increased the proportion of whole body fat utilization during exercise although body mass was not significantly reduced. Also it has been shown that short-term consumption of EGCG compared to placebo increased maximal oxygen uptake in both males and females [44]. The effect of short and longer GTE ingestion on cycling endurance appears to be negligible, however, as both 6 day [45] and 3 week ingestion [46] of GTE did not result in an increase in time trial performance.

Following steady state exercise a significant increase in fat oxidation rate compared to the pre-exercise fasting state has been found [47]. During intense sprinting exercise glycogen stores suffer greater depletion than steady state exercise [48]. Thus, the post-exercise period after ISE should demonstrate enhanced lipid oxidation so that remaining carbohydrates can be utilized for glycogen resynthesis [34]. Results of the present study support this notion as in the placebo condition greater fat oxidation occurred during the last 30 min of post-exercise. McGarvey *et al.* [49] have also shown that when total work was similar, intermittent compared to steady state exercise, resulted in significantly greater fat oxidation during a 2-h post-exercise period. Muscle glycogen response to ISE, however, was not assessed which is also a limitation of the current study. Results of the present study also show that when ISE is accompanied by short term GTE ingestion then fat oxidation is increased throughout post-exercise. During post-exercise the average increase in fat oxidation was 29% after GTE ingestion compared to placebo. The monitoring of metabolic response over a 1-h post-exercise period, however, is a limitation of this study as it is feasible that post-exercise oxygen consumption could continue to occur many hours after exercise [24]. Thus, studies monitoring metabolic and hormonal response to ISE for an extended period are needed.

The effect of gender on the oxidation of triglycerides during ISE recovery does not appear to have been examined, however, a number of studies have assessed fat oxidation response of males and females during aerobic exercise. Unfortunately, gender affects are unclear because of conflicting results. The majority of studies that assessed markers of lipolytic rate during moderate-intensity endurance exercise using microdialysis probes or isotope tracers, found that lipolytic rate markers in females were larger than in males [50,51]. Others, however, found that the lipolytic response to exercise was similar for both genders [52]. Also studies that used indirect calorimetry to assess substrate oxidation have demonstrated that women oxidize more fat and less carbohydrate than men during aerobic exercise [53]. The reasons for these equivocal results is unclear but may involve differences in body composition, aerobic fitness levels, and exercise modality as these factors have been found to influence the rate of lipolysis and fat oxidation during endurance exercise [54,55]. Interestingly, catecholamine levels increase more in males than females during high-intensity exercise [56] which is likely a result of the larger male muscle mass which generates more power and enables men to typically work at a higher intensity than females. Consequently, future research is required to examine the effect of short term green tea ingestion and acute ISE on fat oxidation response of males.

The green tea capsules ingested by participants contained 20 mg of caffeine which was reported by the manufacturer. That the amount of caffeine in the capsules was not verified is a limitation of the study. The effect of caffeine on resting energy expenditure has been examined and studies have shown that a single oral dose of more than 100 mg caffeine is needed to elicit a significant increase in thermogenic response. Also to increase energy expenditure a 600 mg to 1000 mg caffeine dose per day appears to be necessary [57,58]. Thus, it is feasible that the small amount of caffeine ingested by participants in the current study is unlikely to have had a significant effect on their resting and post-exercise fat oxidation levels.

It was predicted that greater fat oxidation would occur after short term ingestion of GTE during the post-exercise period. The increase in post-exercise fat oxidation being driven by enhanced norepinephrine and epinephrine release during ISE causing an increased accumulation of circulatory sulfo-conjugated catecholamines [59]. In contrast to the short half-life of catecholamines (1–3 min) the half-life of sulfo-conjugated catecholamines has been estimated to be 3–4 h [60]. It is thought that green tea catechins increase fat oxidation through inhibition of catechol-*O-*methyltransferase, the enzyme that degrades norepinephrine, thereby prolonging adrenergic drive [15,16,17]. As blood catechin, caffeine, and flavonoid levels were not assessed, however, it is unclear to what extent these nutrients found in green tea affected fat oxidation. The increased norepinephrine and glycerol levels during post-exercise after the GTE ingestion supports the notion that catechins may have made some contribution towards the increase in fat oxidation. Future research should attempt to identify the contribution of catechins, caffeine, and flavonoids to the green tea fat oxidation affect. That epinephrine levels were also elevated after GTE consumption during exercise also suggests that GTE may increase adrenergic drive. However, no relationship was found between adrenergic drive and blood catecholamine level during a 60-min bout of acute aerobic exercise performed at 56% of maximal oxygen uptake after 7 days of GTE ingestion [60]. In a recent review Hodgson *et al.* [61] have pointed out that the inhibition of catechol-*O-*methyltransferase hypothesis has little in vivo support and they suggest that changes in the expression of fat metabolism genes could be brought about with chronic exercise training. The expression of fat metabolism genes could include the upregulation of skeletal muscle fat metabolism enzyme gene and down regulation of hepatic adipogenic gene expression [61]. As it is unlikely that the combination of short term ingestion of GTE and one bout of ISE could bring about transcriptional activity changes more studies have to be carried out in order to identify the mechanisms underlying the short term GTE ingestion and acute ISE fat oxidation effect.

Fifteen weeks of ISE resulted in a significantly greater reduction of subcutaneous fat compared to 15 weeks of steady state cycle exercise [22]. A similar protocol with overweight males also resulted in significant decreases in total and visceral fat [23] and in overweight females a significant decrease in total body fat and central abdominal fat was found [62]. Consequently, the significant elevation in fat oxidation caused by one bout of ISE together with GTE ingestion suggests that repeated use of this combination may have the potential to reduce fat mass of overweight females.

## 5. Conclusions

In conclusion, it was found that short term green tea ingestion significantly elevated fat oxidation and plasma glycerol levels before and post intermittent sprinting exercise. Epinephrine levels were elevated during exercise and norepinephrine levels were increased post-exercise after green tea ingestion.
